# Effect of 8-week intake of the *n*-3 fatty acid-rich perilla oil on the gut function and as a fuel source for female athletes: a randomised trial

**DOI:** 10.1017/S0007114522001805

**Published:** 2023-03-28

**Authors:** Aki Kawamura, Ken Nemoto, Masaaki Sugita

**Affiliations:** Faculty of Sport Science, Nippon Sport Science University, Tokyo 1588508, Japan

**Keywords:** Gut microbiota, *n*-3 unsaturated fatty acids, Female athlete, Volleyball player, Constipation score

## Abstract

Previous studies have examined the effects of *n*-3 fatty acid intake in supplement form or fish oil capsules, but there are few studies based on other foods. Perilla oil is a traditional Japanese seed oil rich in *n*-3 fatty acids. This randomised trial aimed to determine the appropriate *n*-3 fatty acid dose through consumption of perilla oil, which improves gut function and microbiota in trained athletes, and the amount of fat fuel required to provide energy to athletes involved in high-intensity training to improve athletic performance. Thirty-six female athletes training six times per week were randomly assigned to three groups according to perilla oil intake: 9 g/d (high oil intake (HOI)), 3 g/d (low oil intake (LOI)) and placebo-supplementation (PLA) groups. The HOI and LOI groups had perilla oil-containing jelly and the PLA group had placebo jelly for 8 weeks. Gut microbiota, constipation score and urinary biochemical index were measured pre- and post-intervention. The spoilage bacteria, *Proteobacteria*, significantly decreased (*P* = 0·036, *d* = 0·53), whereas *Butyrate*-producing bacteria, *Lachnospiraceae*, significantly increased (*P* = 0·007, *d* = 1·2) in the HOI group. Urinary indoxyl sulphate significantly decreased in the HOI group only (*P* = 0·010, *d* = 0·82). Changes in the constipation score were significantly lower in the HOI group (*P* = 0·020) and even lower in the LOI group (*P* = 0·073) than in the PLA group; there were significant differences between groups (*P* = 0·035). Therefore, perilla oil intake may improve gut function and microbiota in athletes, with higher doses resulting in further improvement.

Physical health is strongly related to the type and number of gut bacteria in hosts. The production of SCFA such as *Butyrate* by health-promoting bacteria fosters immunomodulatory effects and health^(1)^. These bacteria are also relevant to athletes’ health and exercise performance^(1–3)^ because the gut microbiome is closely linked to organ functions, such as the brain^(4–6)^ and muscle^(7,8)^. Metabolites from gut bacteria, such as SCFA, modulate signalling^(9)^; activate metabolic pathways^(10–12)^ and insulin sensitivity^(13)^, particularly in skeletal muscle metabolism; and may affect the control of body weight and exercise performance^(2,12)^. SCFA are produced by gut microbiota, such as *Bacteroidetes* and *Lachnospiraceae* ^(14)^. It has been suggested that *Bacteroidetes* are increased from exercise and gut bacteria, and exercise adaptations may play a role^(15–17)^. Among SCFA, *Butyrate* has been shown to be a key modulator of energy metabolism and mitochondrial function by activating PGC-1*α* gene expression in skeletal muscles and brown adipose tissue^(18)^. The study has also demonstrated that dietary *Butyrate* supplementation improves insulin sensitivity and increases energy expenditure by enhancing mitochondrial function in animals^(18)^. Moreover, increasing bacterial diversity is important for improving adaptability to external stimuli, such as environment and exercise. Furthermore, the diversity of gut bacteria in athletes is higher than that of the general public, which suggests that gut bacteria are adapted to stimulation by exercise and training^(19)^. Taken together, the diversity of gut microbiota and exercise-induced bacteria, such as *Butyrate*-producing bacteria, would be beneficial for athletes who engage in high-intensity training.

The gut is highly adaptable to external factors, such as lifestyle and environmental stimuli. The composition and diversity of gut bacteria are affected by the genetic elements (age, sex and birth route^(20,21)^) and external factors (diet^(22)^, exercise^(23)^ and antibiotics^(24)^). In particular, diet strongly influences the gut microbiome, and this change is caused by long-term dietary patterns^(22)^ and short-term interventions of several weeks^(25,26)^. For many athletes, carbohydrates are the main energy source to maintain performance and recover glycogen stores^(27,28)^. Recent studies have shown that increased intake of carbohydrates in the form of dietary fibre is associated with an increase in the diversity of gut bacteria^(1,29)^. Fats are also important substrates for energy metabolism. Although previous studies have reported that a high-fat diet reduces the diversity of gut bacteria and increases the *Firmicutes* ratio^(30,31)^, the effects of a small amount of fat remain unclear. Therefore, an effective low-dose fat intake strategy is warranted to support fuelling in athletes, especially during high-intensity training periods.

There is inconsistent evidence about the effect of fat intake on the gut microbiome and functions in human and animals. In terms of types of fats, fish oil and unsaturated fatty acid intake increased probiotics, such as *Bifidobacterium* and *Lactobacillus* ^(32,33)^. Saturated fat acid does not increase these bacteria^(33)^. In addition, a high saturated fat diet reduces bacterial numbers and increases the excretion of SCFA^(34)^. A recent review concluded that the *n*-3 fatty acid favours the *Butyrate*-producing bacterial genera, whereas a saturated fat-rich diet can attenuate the gut microbiota of these commensal bacteria^(35)^. Moreover, the effect of fat intake on gut microbiota depends on the type of fatty acid. *n*-3 fatty acids provide multiple health benefits, such as lowering blood pressure^(36)^ and preventing diseases ^(37–39)^, including inflammatory bowel disease^(40)^. It also has several benefits on exercise, including post-exercise muscle recovery ^(41–43)^, training-induced muscle strength^(44)^, reduced muscle loss and inflammation ^(45,46)^, endurance ability^(47)^ and brain health^(48)^. In addition, *n*-3 fatty acids play an important role in physiological adaptation to produce metabolites through cell receptors ^(49,50)^.

Perilla oil is rich in *α*-linolenic acid, a type of *n*-3 fatty acid, which also contains small amounts of linoleic acid of *n*-6 fatty acid and oleic acid of *n*-9 fatty acid. These fatty acids have different properties, but through the intake of perilla oil, a combination of the benefits of these acids can be obtained. Perilla oil contains a large amount of *n*-3 fatty acids not found in other seed oils such as olive oil and maize oil, which are mainly composed of *n*-6 and *n*-9 fatty acid and have extremely low amounts of *n*-3 fatty acids. Furthermore, perilla oil is a traditional Japanese food that can be consumed daily – a notable strength as a research food in this study.

Since the gut environment is correlated with organ function, daily *n*-3 intake may enhance the function of other organs through the improvement of the gut environment. Athletes are required to adapt to muscle and other organ functions at a high level. Therefore, strategies for improving the gut environment to efficiently metabolise nutrients are required. Although previous studies have investigated the effects of *n*-3 fatty acids on the gut environment in animals, healthy humans and patients^(35)^, we hypothesised that *n*-3 fatty acid supplementation could also improve the gut function of athletes. We aim to find an effective use of perilla oil that fuels energy and improves gut function in athletes and evaluate different dose-dependent effects.

## Experimental methods

### Participants

Thirty-six female athletes belonging to a university volleyball club (age: 20·2 (s e 1·3) years, height: 167·8 (s e 7·8) cm, body weight: 63·4 (s e 6·6) kg) were recruited. All participants trained six times a week, an average of 4·5 h per day. This study was conducted according to the guidelines laid down in the Declaration of Helsinki and all procedures involving participants were approved by the ethics committee of Nippon Sport Science University (No. 018-H193) and the University Hospital Medical Information Network Clinical Trials Registry in Japan (No. UMIN000044882). All participants signed a written consent form after being informed on the purpose of the study, methods, possible health hazards, risks, privacy protection, data management and publication. None of the participants was not using supplements and medicines, and history of chronic disease and smoking. The recruitment, data collection and follow-up were conducted from October 2019 to December 2020. All data were collected pre-, during and post-intervention at the Nippon Sport Science University.

Body composition was measured using bioelectrical impedance analysis (InBody730, InBody Co., Ltd.), and the physical characteristics of participants are shown in Table 1. All participants live in university dormitories and daily meals are provided by a dietitian. Since the participants competed in the same sports club and trained six times a week in same training menus, there was no difference in the training load between participants and phases during the intervention period.

**Table 1.** Physical characteristics of participants in pre- and post-intervention (Mean values with their standard errors)

Physical characteristics	HOI						LOI						PLA					
	Pre		Post		Change		Pre		Post		Change		Pre		Post		Change	
	Mean	se	Mean	se	Mean	se	Mean	se	Mean	se	Mean	se	Mean	se	Mean	se	Mean	se
Body weight (kg)	64·1	1·6	63·5	1·6	-0·6	0·3	65·1	2·6	65·6	2·5	0·5	0·2	61·4	1·4	61·2	1·4	- 0·1	0·3
BMI (kg/m^2^)	22·7	0·3	22·5	0·3	-0·2	0·1	22·7	0·4	22·9	0·4	0·2	0·1	21·7	0·4	21·6	0·4	0·0	0·1
Body fat (%)	22·4	1·0	21·7	1·0	-0·7	0·4	21·1	0·7	21·2	0·8	0·1	0·5	21·4	1·6	21·0	1·6	-0·4	0·4
Skeletal muscle mass (kg)	27·7	0·8	27·7	0·8	0·0	0·2	29·3	1·3	29·4	1·3	0·2	0·2	26·2	0·8	26·4	0·8	0·2	0·1
*α*-Diversity	3·96	0·11	4·19	0·14	0·23	0·13	4·46	0·11	4·18	0·14	- 0·29	0·14	4·22	0·12	4·39	0·15	0·17	0·13

*n* 12. HOI: 9 g/d perilla oil intake group, LOI: 3 g/d perilla oil intake group, PLA: placebo-intervention, Pre: pre-intervention, Post: post-intervention.

### Experiment design

**Fig. 1.**
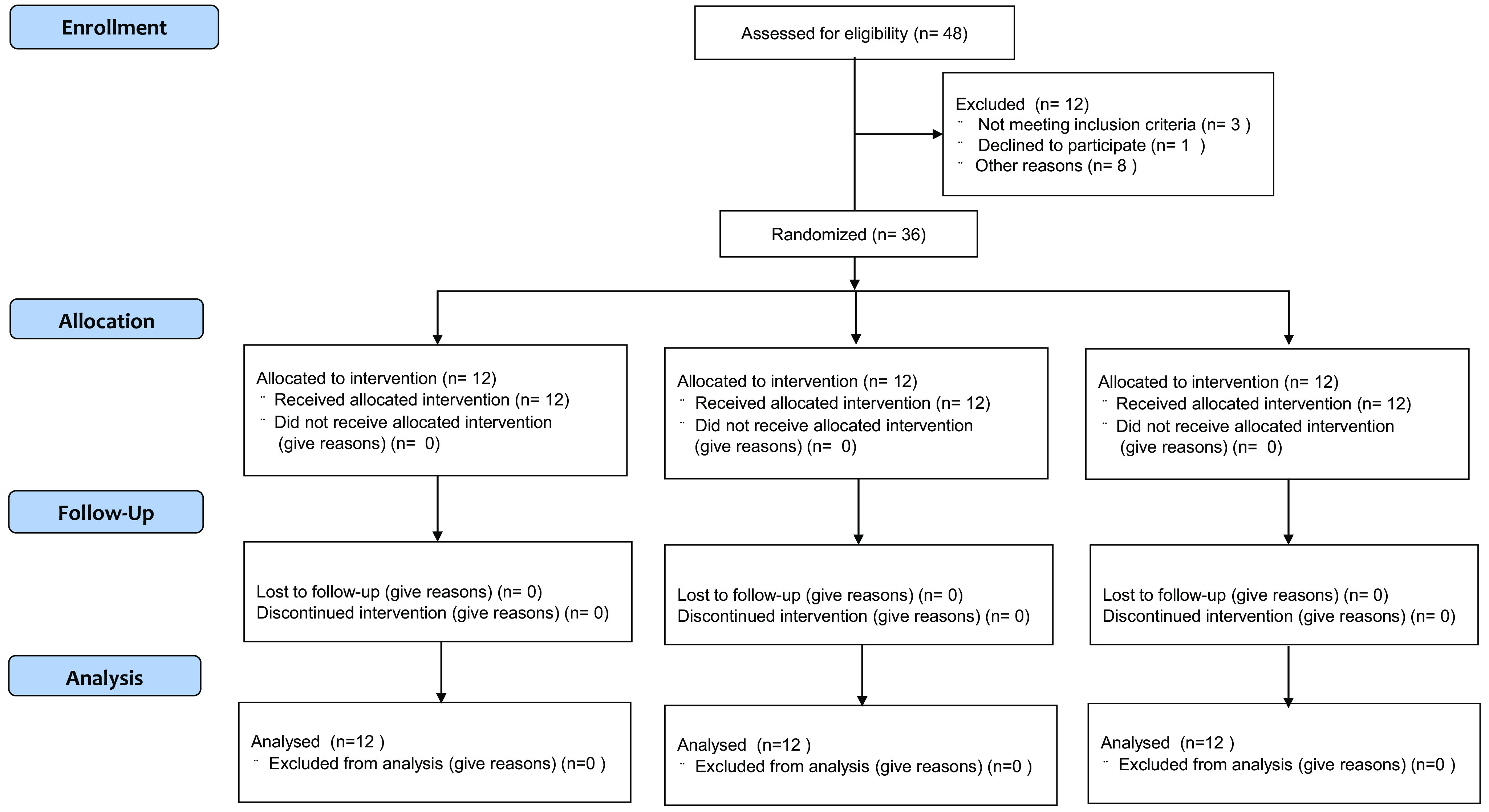
CONSORT flow diagram for the randomised controlled trials.

Forty-eight participants were eligible for this randomised trial, of whom twelve were excluded from the trial: three of them did not meet the criteria, one declined to participate and eight were not allowed to undergo intervention owing to physical reasons. Finally, thirty-six participants were involved in the trial and equally divided into three groups by a third party according to their perilla oil intake as follows: high oil intake (HOI) (9 g/d), low oil intake (LOI) (3 g/d) and placebo-supplementation (PLA) groups (Fig. 1). The participants were blinded to their groupings, which were concealed by sequential numbers. The HOI group received 3 g of perilla oil-containing jelly three times per day (9 g/d of perilla oil), while the LOI group received 3 g of perilla oil-containing jelly once per day (3 g/d of perilla oil). The PLA group ingested a jelly with the same shape and taste as perilla oil-containing jelly once per day (0 g/d of perilla oil) during the intervention period. Subsequently, we compared the effects of high doses with those of the generally recommended dose of *n*-3 fatty acids for athletes ^(51,52)^ and placebo intake. Participants were instructed to maintain their diet, training or lifestyle during the intervention period. We referred to a previous double-blinded, randomised, controlled study examining the effects of nutrient intake on athletes’ gut microbiota to determine the number of participants^(53)^.

**Fig. 2.**
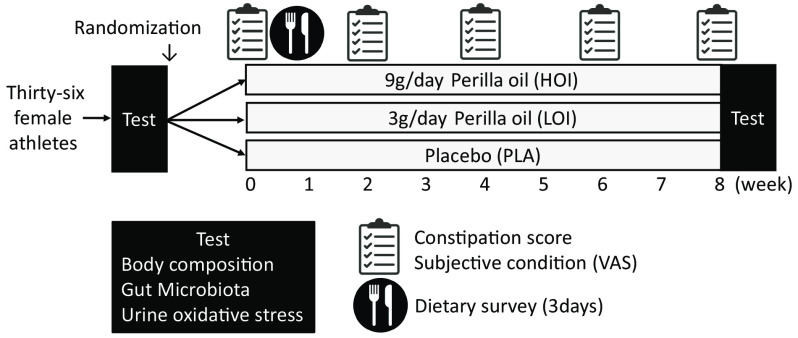
Experiment design.

We investigated body weight, body composition, gut microbiota and urinary biochemical index (indoxyl sulphate, 8-hydroxydeoxyguanosine:8-OHdG). In addition, data on constipation score, subjective condition questionnaire about fatigue, sleep quality, appetite, psychological stress and training load using the visual analogue scale method and sleep hours were obtained pre-intervention and every 2 weeks thereafter (Fig. 2).

### Faecal microbiota

Bacterial DNA from faecal samples was collected in a solution containing 4 M guanidine thiocyanate for analysis. Faecal samples were pre-treated with zirconia beads, followed by DNA extraction and purification using an automated DNA extraction and purification system (Maxwell®, Promega Corporation). 16S rRNA bacterial primers containing the V1 and V2 regions – targets for determining the species^(54,55)^ – were prepared. The following cycling conditions were employed: 10 s at 98°C, 10 s at 55°C and 5 s at 72°C for 20 cycles. Then, the samples were subjected to next-generation sequencing, and approximately 300 bases, including the V1–V2 variable regions, were analysed as described previously^(54)^. The number of effective reads per sample was approximately 30 000–100 000. Then, the *α*-diversity (Shannon index) was determined and bacteria were identified. Diversity analysis was performed using an Excel add-on (Ekuseru-Toukei 2015, Social Survey Research Information Co., Ltd.), and the outcomes were presented using Shannon indexes. All phylum-, family- and genus-level changes were analysed. Detection and analysis of faecal bacteria were outsourced (SheepMedical Co., Ltd.).

### Constipation score

A subjective questionnaire by Agachan *et al.* ^(56)^ was used to assess constipation status during the intervention period (pre-intervention and every 2 weeks thereafter). The survey included frequency of bowel movements, difficulty of defecation, abdominal pain, time required for the laboratory, need for assistance, number of failures and history of constipation. The total scores ranged from 0 to 30, with 0 indicating no constipation and 30 indicating severe constipation. The change in score was calculated from the pre-value minus the lowest value during the intervention.

### Urinary biochemical index

Indoxyl sulphate is a gut-derived uremic toxin mainly produced from tryptophan-containing foods, such as egg white, meat, milk, cheese and soya product^(57)^. 8-OHdG is a biomarker of oxidative DNA damage^(58)^. These indicators were outsourced for analysis (Healthcare Systems Co., Ltd.).

### Perilla oil supplementation and nutrient intake

Perilla oil is a traditional Japanese seed oil that contains high levels of *n*-3 fatty acids. Perilla oil-containing jelly was provided to the HOI and LOI group participants, and placebo jelly was provided to the PLA group participants. Both jellies were lemon-flavoured, obscuring the natural nutty taste of perilla oil. Participants in the HOI group ingested one jelly after breakfast, lunch and dinner, whereas those in the LOI and PLA group ingested one jelly after lunch. Nutritional components per jelly and fatty acid composition in perilla oil are shown in Tables 2 and 3.

**Table 2.** Nutritional components of perilla and placebo jelly per one portion

Energy and nutrients	Perilla oil jelly	Placebo jelly
	(20 g)	(20 g)
Energy (kJ) (kcal)	9 (38)	3 (12)
Protein (g)	0·0	0·0
Fat (g)	3·0	0·0
SFA (g)	0·0	0·0
Unsaturated fatty acids (g)	3·0	0·0
Carbohydrate (g)	2·8	3·3
Salt (g)	0·08	0·10

**Table 3.** Fatty acid composition in perilla oil

Fatty acid		Ratio (%)
*n*-3	*α*-Linolenic acid	62·6
*n*-6	Linoleic acid	15·4
*n*-9	Oleic acid	13·2
Others		7·9

A dietary assessment was conducted using a dietary record maintained for 3 consecutive days to calculate participants’ nutrient intake pre-intervention. All participants could eat freely during the intervention, and their food intakes were recorded using a food diary and camera to click food pictures. Then, a dietitian reviewed their diet and estimated participants’ energy, macronutrient intake, dietary fibre, vitamins and minerals using a software (NEW HEALTHY ver. Tokyo Shoseki Co., Ltd.).

### Subjective condition

The degree of subjective conditions on fatigue, sleep quality, appetite, psychological stress and training load was measured using the visual analogue scale method. The participants were asked to indicate the degree of subjective on a 100-mm horizontal line. The left side (0 mm) indicated ‘having bad condition’, whereas the right side (100 mm) showed ‘having good condition’.

### Statistical analysis

All data are reported as mean values with their standard error. Differences within groups and between groups were determined using the paired *t* test and unpaired *t* test or ANCOVA, respectively. When significant differences were determined using ANCOVA, post hoc analyses were conducted using the Bonferroni test. For parameters with skewed distribution, the Kruskal–Wallis test was performed for comparison of the three groups. Cohen’s *d* was calculated to measure the effect size. Statistical analysis was performed using SPSS ver.25 (IBM Japan Inc.), and *p* values < 0·05 were considered statistically significant.

## Results

The participants were excellent adherence to the intervention with no dropouts.

### Body composition

There were no significant differences in body weight (kg), BMI (kg/m^2^), body fat (%) and skeletal muscle mass (kg) between pre- and post-intervention in all groups (Table 1). Habitual sleep hours (h:min) at pre- and post-intervention were 6:46 (se 0:12) and 6:30 (se 0:11) in the HOI group, 6:39 (se 0:23) and 6:50 (se 0:23) in the LOI group and 6:58 (se 0:22) and 6:50 (se 0:13) in the PLA group, respectively, with no difference between the groups.

### Faecal microbiota

**Fig. 3.**
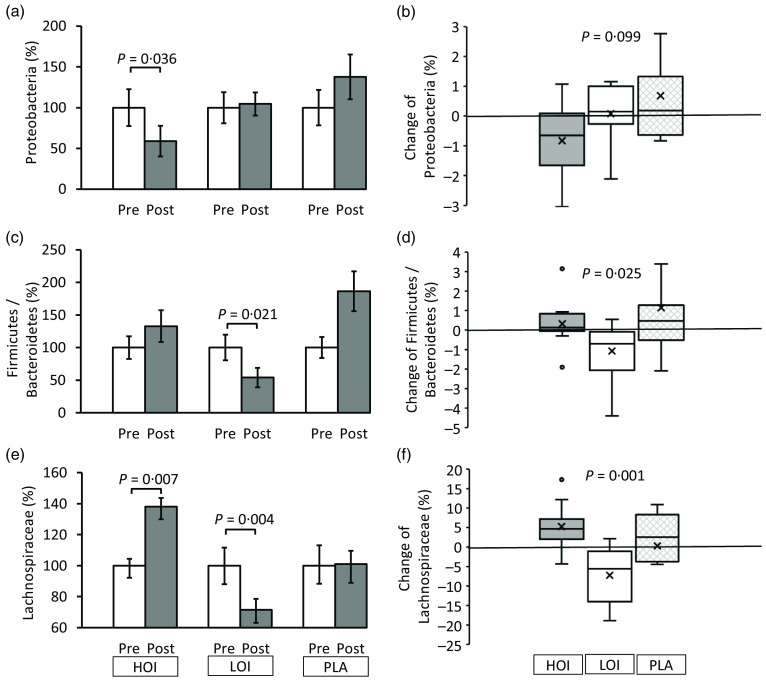
Changes in faecal microbiota pre- and post-intervention at the phylum and family level. (a), (c), (e) Comparison of the faecal microbiota within and between the groups. (b), (d), (f) Changes of the faecal microbiota between the groups. Data are presented as the mean values with their standard error, minimum and maximum, *n* 12. *P*-value; differences pre- and post-intervention within the group or difference of the changes between groups, HOI: 9 g/d perilla oil intake group, LOI: 3 g/d perilla oil intake group, PLA: placebo-intervention, Pre: pre-intervention, Post: post-intervention.

The *α*-diversity pre- and post-intervention changed from 3·96 (se 0·11) to 4·19 (se 0·14) in the HOI group (*P* = 0·147), 4·46 (se 0·11) to 4·18 (se 0·14) in the LOI group (*P* = 0·107) and 4·22 (se 0·12) to 4·39 (se 0·15) in the PLA group (*P* = 0·955). No differences were observed between the groups. Regarding bacterial changes at the phylum level, the spoilage bacteria, *Proteobacteria*, significantly decreased post-intervention (1·2 (se 0·2)) compared with those pre-intervention (2·0 (se 0·5)) in the HOI group (*P* = 0·036, *d* = 0·53). There was no change between pre- and post-intervention in the LOI and PLA groups. The change tended to be different among the three groups (*P* = 0·099). *Firmicutes* were significantly decreased post-intervention (47·2 (se 4·0)) compared with those pre-intervention (56·5 (se 4·5)) in the LOI group (*P* = 0·002, *d* = 0·59). In contrast, *Bacteroidetes* were significantly increased post-intervention (43·2 (se 3·7)) compared with those pre-intervention (31·6 (se 3·8)) in the LOI group (*P* = 0·004, *d* = 0·89). The changes in these bacteria were significantly different between the groups (*P* = 0·016). Additionally, the *Firmicutes*/*Bacteroidetes* (F:B) ratio was significantly decreased post-intervention (1·3 (se 0·2)) compared with that pre-intervention (2·3 (se 0·5)) in the LOI group (*P* = 0·021, *d* = 0·68). The changes were significantly different between the groups (*P* = 0·025). For the bacterial changes at the family level, *Butyrate*-producing bacteria, *Lachnospiraceae*, were significantly increased post-intervention (19·0 (se 1·6)) compared with those pre-intervention (13·8 (se 1·3)) in the HOI group (*P* = 0·007, *d* = 1·2). In contrast, they were significantly decreased post-intervention (18·2(se 2·0)) compared with those pre-intervention (25·5 (se 3·4)) in the LOI group (*P* = 0·004, *d* = 0·62) and did not change in the PLA group. The change was significantly different among the three groups (*P* = 0·001) (Table 4, Fig. 3).

**Table 4.** The relative abundance of faecal bacteria pre- and post-intervention at the phylum and family level (Mean values and standard deviations)

Level	Bacteria	HOI							LOI							PLA							*P* †
		Pre		Post		Change		*P* *	Pre		Post		Change		*P* *	Pre		Post		Change		*P* *	
		Mean	sd	Mean	sd	Mean	sd		Mean	sd	Mean	sd	Mean	sd		Mean	sd	Mean	sd	Mean	sd		
Phylum	*Proteobacteria*	2·0	0·5	1·2	0·2	–0·8	0·3	0·036	1·7	0·3	1·8	0·3	0·1	0·3	0·794	1·8	0·4	2·5	0·7	0·7	0·8	0·415	0·099
	*Firmicutes*	42·0	3·3	47·8	3·2	5·8	3·9	0·182	56·5	4·5	47·2	4·0	–9·2	3·3	0·002	55·1	4·2	61·3	3·6	6·2	4·4	0·205	0·025
	*Bacteroidetes*	49·9	3·9	46·4	3·8	–3·5	4·7	0·492	31·6	3·8	43·2	3·7	11·6	3·1	0·004	37·9	4·4	31·5	3·8	–6·5	4·9	0·233	0·016
	*Firmicutes/Bacteroidetes*	1·0	0·2	1·3	0·3	0·3	0·3	0·354	2·3	0·5	1·3	0·2	–1·1	0·4	0·021	1·4	0·2	2·7	0·8	1·1	0·8	0·203	0·025
	*Other*	6·1	1·7	4·6	1·1	–1·5	1·7	0·420	10·2	2·7	7·8	2·2	–2·5	2·5	0·368	5·1	1·3	4·7	1·1	–0·4	1·0	0·678	0·973
Family	*Bifidobacteriaceae*	5·5	1·6	3·5	0·8	–2·0	1·8	0·299	9·5	2·7	6·9	2·3	–2·7	2·5	0·331	4·7	1·3	4·4	1·1	–0·3	1·0	0·797	0·758
	*Bacteroidaceae*	38·3	4·3	36·9	4·1	–1·5	4·2	0·747	22·6	4·0	30·1	5·0	7·5	1·8	0·052	34·3	4·6	28·1	3·5	–6·2	4·9	0·248	0·129
	*Porphyromonadaceae*	3·4	0·7	3·3	0·8	–0·1	0·6	0·858	2·7	0·6	2·8	0·5	0·1	0·5	0·846	2·6	0·9	1·6	0·4	–1·0	0·7	0·174	0·330
	*Prevotellaceae*	7·3	3·7	5·0	4·0	–2·3	2·7	0·441	5·0	2·2	9·4	4·5	4·3	2·5	0·122	0·7	0·3	1·3	0·7	0·6	0·6	0·356	0·122
	*Streptococcaceae*	4·2	1·9	1·9	0·6	–2·3	1·9	0·266	2·9	1·0	2·0	0·5	–0·9	0·9	0·376	1·8	0·3	2·4	0·7	0·6	0·7	0·429	0·913
	*Clostridiaceae*	1·3	0·2	2·4	0·5	1·1	0·5	0·074	2·4	0·8	1·4	0·2	–1·0	0·7	0·208	2·0	0·4	3·8	0·9	1·8	1·0	0·100	0·032
	*Eubacteriaceae*	2·3	0·7	3·1	1·0	0·8	0·6	0·274	3·3	0·7	3·0	1·0	–0·2	0·9	0·844	3·7	0·7	4·7	1·1	1·0	0·4	0·060	0·159
	*Lachnospiraceae*	13·8	1·3	19·0	1·6	5·2	1·5	0·007	25·5	3·4	18·2	2·0	–7·3	2·0	0·004	28·7	3·8	29·0	2·5	0·3	2·8	0·927	0·001
	*Ruminococcaceae*	14·8	2·2	14·2	2·3	–0·6	1·9	0·769	14·4	2·9	15·2	3·6	0·7	2·3	0·761	11·8	2·9	14·5	2·0	2·6	2·7	0·367	0·420
	*Other*	9·1	1·4	10·7	1·6	1·6	1·6	0·338	11·6	1·3	11·0	1·4	–0·6	1·1	0·620	9·6	1·8	10·2	1·1	0·6	1·7	0·737	0·558
Genus	*Faecalibacterium*	11·1	2·2	7·9	2·1	–3·2	1·2	0·027	8·2	1·8	11·5	3·3	3·4	2·0	0·136	7·4	1·1	11·0	1·7	3·6	1·9	0·098	0·011
	*Bacteroides*	38·3	4·3	36·9	4·1	–1·5	4·2	0·747	22·6	4·0	30·1	5·0	7·4	3·3	0·052	34·3	4·6	28·1	3·5	–6·2	4·9	0·248	0·134
	*Eubacterium*	3·7	1·0	4·4	1·1	0·7	0·6	0·310	7·8	1·8	5·0	1·4	–2·8	1·1	0·033	8·5	2·6	9·2	1·8	–0·7	2·1	0·759	0·024

*n* 12. HOI: 9 g/d perilla oil intake group, LOI: 3 g/d perilla oil intake group, PLA: placebo-intervention, Pre: pre-intervention, Post: post-intervention.

* Differences pre- and post-intervention within the groups.

† Differences in changes between the groups.

### Constipation score

**Fig. 4.**
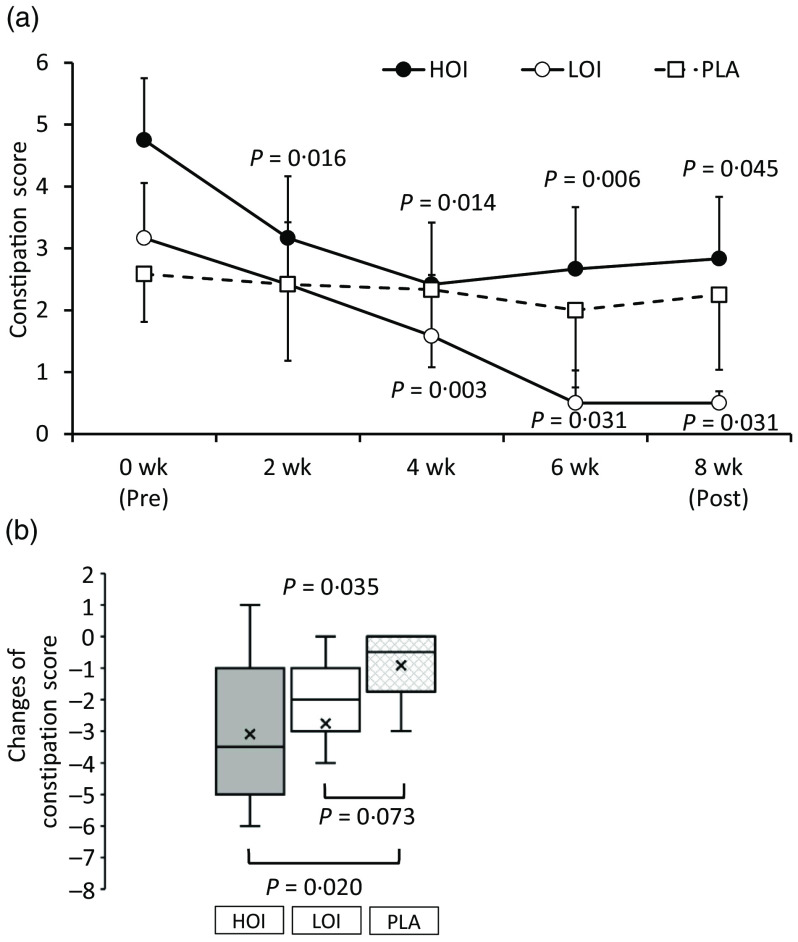
Comparison of the constipation score between the groups. (a) Constipation score during intervention period. (b) Changes of the constipation score between the groups. Data are presented as the mean values with their standard error, minimum and maximum, *n* 12. *P*-value; differences pre- and during/post-intervention within the group, differences compared with PLA or differences between groups, HOI: 9 g/d perilla oil intake group, LOI: 3 g/d perilla oil intake group, PLA: placebo-intervention, Pre: pre-intervention, Post: post-intervention.

The constipation score was significantly decreased during the intervention period in the HOI and LOI groups; however, there was no change in the PLA group. The change was significantly lower in the HOI group (*P* = 0·020) and tended to be lower in the LOI group (*P* = 0·073) than that in the PLA group (Fig. 4). Changes were significantly different between the groups (*P* = 0·035).

### Urinary biochemical index

**Fig. 5.**
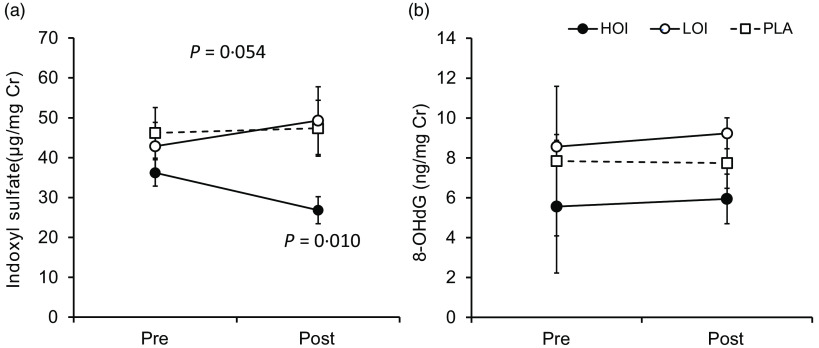
Changes in urinary biochemical index pre- and post-intervention. (a) Comparison of indoxyl sulphate within and between the groups. (b) Comparison of 8-OHdG within and between the groups. Data are presented as the mean values with their standard error, *n* 12. *P*-value; differences pre- and post-intervention within the group or difference of the changes between groups.

Indoxyl sulphate (µg/mg Cr), which is an indicator of the deterioration of the intestinal environment, was significantly decreased post-intervention (26·8 (se 3·4)) compared with that pre-intervention (36·2 (se 3·3)) in the HOI group (*P* = 0·010, *d* = 0·82). The change tended to be different among the three groups (*P* = 0·054). 8-OHdG, a biomarker for oxidative damage of DNA, did not change between pre- and post-intervention in all groups (Fig. 5).

### Perilla oil supplementation and nutrient intake

Harms or unintended effects by perilla oil supplementation were not reported.

The daily increase in energy intake through consumption of the jelly was 114, 38 and 12 kcal in the HOI, LOI and PLA groups, respectively. The intake of unsaturated fatty acids, *n*-3 fatty acid and *n*-6 fatty acid acids by groups did not differ significantly before the intervention. Similarly, the intake of total energy, fat, carbohydrate, fibre, vitamins and minerals by the groups did not differ significantly before the intervention (Table 5). Notably, none of the participants changed their eating habits during the intervention period.

**Table 5.** Daily intake of nutrients pre-intervention (Mean values with their standard errors)

	HOI		LOI		PLA	
Energy and nutrients	Mean	se	Mean	se	Mean	se
Energy (kJ/d) (kcal/d)	638 (2671)	12 (51)	660 (2762)	15 (62)	662 (2771)	5 (21)
Protein (g/d)	111·8	8·1	106·5	5·8	108·6	3·9
Fat (g/d)	108·0	9·8	117·9	2·6	115·9	2·4
SFA (g/d)	27·1	4·1	31·6	1·0	30·9	1·3
Unsaturated fatty acids (g/d)	25·3	0·6	27·7	1·7	27·5	1·3
*n*-3 PUFA (g/d)	4·0	0·6	3·5	0·3	3·5	0·2
*n*-6 PUFA (g/d)	21·2	0·4	24·2	1·5	24·0	1·2
Carbohydrate (g/d)	297·8	18·4	302·6	7·7	307·1	5·2
Total dietary fibre (g/d)	18·2	2·3	17·7	1·8	17·2	2·2
Water-soluble dietary fibre (g/d)	4·6	0·8	4·4	0·4	4·2	0·6
Insoluble dietary fibre (g/d)	13·2	1·4	13·1	1·3	12·8	1·5
K (mg/d)	3489·7	250·2	3339·3	191·2	3320·0	182·7
Ca (mg/d)	733·3	18·4	581·0	45·6	583·3	57·8
Mg (mg/d)	442·7	27·1	422·7	25·9	436·7	39·8
Fe (mg/d)	13·7	1·1	12·3	0·5	12·3	0·5
Zn (mg/d)	13·9	2·6	14·5	1·3	14·3	1·4
Vitamin A (µg/d)	503	99	676	58	618	82
Vitamin D (µg/d)	3·9	1·3	5·1	1·0	5·1	0·9
Vitamin E (mg/d)	9	0·7	10	0·3	9	0·2
Vitamin K (µg/d)	592·3	120·9	660·0	98·7	640·0	105·6
Vitamin B_1_ (mg/d)	1·4	0·2	1·6	0·3	1·5	0·3
Vitamin B_2_ (mg/d)	1·9	0·2	1·9	0·0	1·9	0·0
Vitamin B_6_ (mg/d)	1·6	0·1	1·6	0·0	1·5	0·0
Folic acid (µg/d)	417·3	51·0	455·7	37·3	436·7	45·6
Vitamin C (mg/d)	123	15·6	116	13·1	112	16·4
Salt (g/d)	14·5	1·0	14·4	0·9	13·2	1·2

*n* 12. HOI: 9 g/d perilla oil intake group, LOI: 3 g/d perilla oil intake group, PLA: Placebo-intervention group, Pre: pre-intervention, Post: post-intervention.

### Subjective condition

There was no significant change in the subjective condition every 2 weeks in all groups (Table 6).

**Table 6.** Subjective condition during the intervention period (Mean values with their standard errors)

Subjective condition	HOI										LOI										PLA									
	0 week		2 weeks		4 weeks		6 weeks		8 weeks		0 week		2 weeks		4 weeks		6 weeks		8 weeks		0 week		2 weeks		4 weeks		6 weeks		8 weeks	
	Mean	se	Mean	se	Mean	se	Mean	se	Mean	se	Mean	se	Mean	se	Mean	se	Mean	se	Mean	se	Mean	se	Mean	se	Mean	se	Mean	se	Mean	se
Fatigue	51·9	6·8	51·5	6·9	46·7	5·9	51·6	7·3	53·1	7·2	56·4	6·7	49·1	3·5	45·1	3·9	48·0	6·2	59·7	6·3	47·5	5·6	45·3	4·9	46·8	5·4	50·2	2·8	56·4	5·5
Sleep quality	61·1	7·7	64·3	8·0	64·2	7·2	73·5	6·5	72·8	8·0	60·4	9·7	62·4	5·9	58·5	6·7	74·1	3·9	67·5	5·9	58·6	6·5	65·0	6·8	65·9	5·5	78·3	4·6	65·8	5·3
Appetite	61·3	6·9	61·3	5·4	73·7	4·9	61·8	6·3	65·0	6·3	60·3	6·8	62·3	5·8	64·2	6·2	62·8	6·0	67·4	5·9	76·8	4·9	79·0	5·2	80·2	5·2	82·1	5·9	84·2	4·8
Psychological stress	61·8	5·3	60·8	5·4	72·0	5·3	70·8	6·1	63·8	6·1	57·8	5·8	60·6	4·7	65·1	6·4	71·3	4·5	68·9	4·6	62·0	3·0	61·6	5·3	61·3	6·2	70·2	3·4	68·2	7·0
Training load	61·1	3·8	47·7	6·0	50·8	5·7	47·5	7·2	50·3	7·8	59·4	9·3	48·9	3·9	55·4	5·4	55·7	5·4	60·1	7·5	52·5	3·6	40·0	3·8	45·3	4·8	49·2	4·3	55·6	5·2

*n* 12. HOI: 9 g/d perilla oil intake group, LOI: 3 g/d perilla oil intake group, PLA: placebo-intervention.

## Discussion

Our study revealed that 8-week 9 g/d *n*-3 fatty acid intake increased the abundance of *Butyrate*-producing bacteria and relieved constipation in trained female athletes. We speculated that the intake of *n*-3 fatty acids increased gut SCFA by increasing *Butyrate*-producing bacteria^(14)^ although SCFA could not be measured in this study. In contrast, 3 g/d of perilla oil decreased *Lachnospiraceae*. This change was considered the result of the degradation of the occupancy rate of butyric acid-producing bacteria by the significant increase in *Bacteroidaceae* at the family level and *Bacteroidetes* at the phylum level. Previous studies have reported that *n*-3 fatty acids cause different changes in the gut microbiota profile according to dose^(59)^. Our study suggested that 9 g/d perilla oil intake was sufficient to increase *Butyrat*e-producing bacteria on trained female athletes, but 3 g/d perilla oil intake was not. Ingestion of 3 g of perilla oil increased the abundance of *Bacteroidetes* and decreased the F:B ratio, which may lead to the production of SCFA. In addition, *Lachnospiraceae* were lower at baseline in the HOI group, and *Bacteroidetes* in the LOI group were lower than that in other groups at baseline, which may have led to the increase of these bacteria due to perilla oil intake. The changes in these bacteria may be related to the gut microbiota of the host at baseline. Taken together, the ingestion of perilla oil is superior in increasing SCFA-producing bacteria.

*Butyrate* and SCFA ameliorate inflammatory bowel disease; however, the mechanism of action remains unelucidated^(60)^. A previous study has shown that functional constipation is associated with altered concentrations of butyric acid in mice^(61)^. Other studies have shown that SCFA stimulate the mucous membrane of the large intestine to promote intestinal peristalsis ^(62,63)^. Therefore, growing *Butyrate* may improve gastrointestinal disorders, such as constipation. Our study revealed that constipation scores relieved 2 weeks after the intervention in the HOI group and 4 weeks after in the LOI group of female athletes. Therefore, increased intake of perilla oil relieved constipation in a short period. The relationship between relieve constipation and gut microbiota remains unelucidated, and our study suggests that *Butyrate*-producing bacteria may contribute to functional gut improvement. In addition, ingestion of 9 g/d perilla oil suppressed the growth of *Proteobacteria*, which are related to gut microbiota disturbance. In addition, the uremic toxin indoxyl sulphate was suppressed in the HOI group, which may have led to the improvement of gut function^(64)^. Although *Proteobacteria* and urinary indoxyl sulphate levels were not different between the groups at baseline, they did not change in the LOI group. Therefore, the dose of perilla oil that suppresses indicators related to gut microbiota disturbance should be investigated in the future. Therefore, perilla oil may improve gut microbiota in athletes, and ingestion of 9 g/d perilla oil that is higher than the recommended dose of *n*-3 fatty acid^(52)^ further improves the gut function.

Regarding functional gut disorders, several cross-sectional studies have shown that females are more likely to report constipation than males^(65,66)^ and nearly half of female athletes who are involved in strenuous exercise have gastrointestinal disorders^(67)^. Gut disorders may impair the absorption of nutrients and cause functional disorders, which leads to performance degradation in athletes. Therefore, increasing *Butyrat*e-producing bacteria may benefit female athletes. It has been reported that stimulation and stress caused by excessive exercise may lead to degradation of the diversity of gut bacteria^(68)^. Since the gut environment of athletes is exposed to exercise-induced excessive stress, the gastrointestinal function of athletes tends to deteriorate. Nevertheless, athletes have a higher diversity of gut bacteria than the general population to adapt to external stimuli^(19)^. This study suggests that perilla oil intake would support to suppress gut stress and constipation in high-intensity trained athletes. However, there was no change in the diversity of gut bacteria, 8-OHdG and subjective conditions during the 8-week intervention in this study. In the future, long-term intervention may change these indicators by improving the gut environment.

Finally, despite the additional energy of 114 kcal/d in the HOI group and 38 kcal/d in the LOI group, body weight and body fat did not change. Therefore, daily intake of *n*-3 perilla oil may support athletes’ fuel intake without unexpected weight gain. A simple method to ensure the intake would be to include three teaspoons of perilla oil (approximately 9 g of oil) to daily diet such as salad, or bread and pasta. Gut microbiota differs depending on race and may be different to Japanese and people from other countries^(69)^. Therefore, perilla oil might be effective in improving gut function and microbiota, at least in Japanese athletes. In addition, perilla oil intake for several weeks or more is desirable to improve constipation. Since the effect on gut microbiota differs depending on the dose of perilla oil, further studies are needed to investigate the appropriate intake. Moreover, it has been shown that ingestion of *n*-3 fatty acid improved muscle function^(44,47)^, in addition to producing metabolites^(35)^. Therefore, daily intake of perilla oil may help improve athletic performance.

Our study showed that the *n*-3 fatty acid-rich perilla oil increased butyric acid-producing bacteria and improved gut function. However, this study has several limitations. First, the HOI group received the intervention three times daily to reach the targeted dose, while the other two groups received it once daily. These three groups did not follow the same intervention protocols. However, the study design was unified except for the variation of intake timing. Second, daily surveys throughout the intervention period could not be conducted because it would tremendously inconvenience participants. Although we conducted a dietary survey for 3 d to confirm that there was no difference in nutrient intake between the groups at baseline, a dietary survey was needed throughout the intervention period to completely eliminate any influence of participants’ daily diet. However, a registered dietitian managed the dormitory diet throughout the intervention period; there was no change in the nutrient intake of participants. Since participants resided together in the dormitory, there was no change in diet, lifestyle and training during the intervention period. Third, we could not directly measure the changes in SCFA levels. Although *Butyrate*-producing bacteria promote the production of SCFA, the effect of perilla oil intake on the change in SCFA should be clarified in future studies.

In conclusion, this study showed that a daily intake of 9 g/d perilla oil enhanced the abundance of *Butyrate*-producing bacteria *Lachnospiraceae* and suppressed that of *Proteobacteria* and urinary indoxyl sulphate levels. This effect was not observed in 3 g/d perilla oil intake group. While, there were improvements in the gut function in both groups. The finer dose of perilla oil that stimulates the production of metabolites from gut bacteria and suppresses gut-disturbance indicators should be investigated in the future. Daily *n*-3 fatty acid intake through consumption of perilla oil would be beneficial for enhancing gut microbiota growth and function as well as a fuel source for trained female athletes.
